# Behavioral Economic Strategies to Improve Enrollment Rates in Clinical Research: Embedded Recruitment Pilot Trial

**DOI:** 10.2196/47121

**Published:** 2023-07-21

**Authors:** Brittney Greene, Leah Bernardo, Morgan Thompson, James Loughead, Rebecca Ashare

**Affiliations:** 1 State University of New York at Buffalo Buffalo, NY United States; 2 Perelman School of Medicine University of Pennsylvania Philadelphia, PA United States; 3 School of Nursing University of Pennsylvania Philadelphia, PA United States

**Keywords:** behavior change, behavioral economics, clinical trials, contingency management, evidence based, information provision, recruitment, retention, SMS text messaging, study within a trial, SWAT

## Abstract

**Background:**

Nearly 1 in 3 clinical trials end prematurely due to underenrollment. Strategies to enhance recruitment are often implemented without scientific rigor to evaluate efficacy. Evidence-based, cost-effective behavioral economic strategies designed to influence decision-making may be useful to promote clinical trial enrollment.

**Objective:**

This study evaluated 2 behavioral economic strategies to improve enrollment and retention rates across 4 clinical trials: information provision (IP) and contingency management (CM; ie, lottery). IP targets descriptive and injunctive norms about participating in research and CM provides participants incentives to reinforce a target behavior.

**Methods:**

A sample of 212 participants was enrolled across 4 clinical trials focused on tobacco use: 2 focused on HIV and 2 focused on neuroimaging. The CM condition included a lottery: for each study visit completed, participants received 5 “draws” from a bowl containing 500 “chips” valued at US $0, US $1, US $5, or US $100. In the IP condition, text messages that targeted injunctive norms about research (eg, “Many find it a rewarding way to advance science and be part of a community”) were sent through the Way to Health platform before all study visits. Participants were randomized to 1 of 4 conditions: IP, CM, IP+CM, or standard recruitment (SR). We performed logistic regression, controlling for sex and study, with condition as a between-subject predictor. Outcomes were the percentage of participants who attended a final eligibility visit (primary), met intent-to-treat (ITT) criteria (secondary), and completed the study (secondary). Recruitment was evaluated by the percentage of participants who attended a final eligibility visit, enrollment by ITT status, and retention by the percentage of participants who completed the study.

**Results:**

Rates of attending the eligibility visit and meeting ITT status were 58.9% (33/56) and 33.9% (19/56) for IP+CM; 45.5% (25/55) and 18.2% (10/55) for IP only; 41.5% (22/53) and 18.9% (10/53) for CM only; and 37.5% (18/48) and 12.5% (6/48) for SR, respectively. In the logistic regression, females were more likely to meet ITT status than males (odds ratio [OR] 2.7, 95% CI 1.2-5.7; *P*=.01). The IP+CM group was twice as likely to attend the final eligibility visit than the SR group (OR 2.4, 95% CI 1.1-5.2; *P=*.04). The IP+CM group was also significantly more likely to reach ITT status than the SR condition (OR 3.9, 95% CI 1.3-11.1; *P=*.01). Those who received any active intervention (IP, CM, or IP+CM) had a higher study completion rate (33/53, 63.5%) compared to those who received SR (5/12, 41.7%), but this difference was not significant (*P*=.26).

**Conclusions:**

Combining IP and CM strategies may motivate participants to participate in research and improve recruitment and retention rates. Evidence from this study provides preliminary support for the utility of behavioral economics strategies to improve enrollment and reduce attrition in clinical trials.

## Introduction

Although clinical trials rely heavily on participant engagement to determine the efficacy and effectiveness of their interventions, nearly 1 in 3 closes prematurely due to underenrollment [1]. Acquiring optimal recruitment and retention presents a challenge to clinical trials, which may take a financial toll, reduce statistical power due to an inadequate number of participants, and hinder overall trial success. Nevertheless, many studies implement recruitment strategies without taking a systematic approach to identifying the most efficient and cost-effective methods for enrolling participants. A study within a trial (SWAT) may offer substantial utility for improving a process by testing whether an intervention is successful before employing the method in a future trial [2,3]. SWATs are embedded within host trials and can increase recruitment and retention by assessing which methodologies are most successful for enrolling and retaining participants [4].

SMS text messaging, which can be used to remind participants about upcoming appointments, may be one strategy to improve recruitment and retention (eg, reminders). In the United States, approximately 80%-90% of adults (18 and over) own a mobile phone, and these rates are observed among individuals in rural areas [5-7]. Given the ubiquity of mobile phones, SMS text messaging may reduce financial burden and cultivate a larger and more adherent population in clinical trials. Recent studies have started to evaluate various SMS text messaging interventions through SWATs, but results have been mixed [8-11]. Among studies evaluating SMS text messaging interventions, retention rates were high (89%-90%), with low attrition commonly cited [9-11]. However, SMS text messaging interventions to improve recruitment also face challenges, including a lack of digital literacy among participants [8,10,12-14]. To address these gaps, recent work has turned to behavioral economics frameworks that may target these recruitment and retention challenges.

Behavioral economics is a field guided by the framework of operant conditioning that examines social and emotional factors that contribute to decision-making [15,16]. Behavioral economic principles have been applied to various disciplines, including policy, health behavior, and psychology, to explain and influence human choice [17-19]. Previous research has incorporated behavioral economic strategies to address enrollment challenges and improve intervention outcomes with variable success. Contingency management (CM) is a behavioral economic technique that provides participants incentives to reinforce a target behavior. Financial incentives have increased participant retention and engagement, particularly in studies with incremental cash costs [20-22]. Lotteries, a common form of CM, allow participants a chance to win some money in exchange for achieving a study milestone (eg, attending a visit and maintaining abstinence from smoking). Although lotteries may reduce financial burden and improve retention, they have demonstrated differential outcomes for behavioral change. Lotteries have improved physical activity, weight loss, and ecological momentary assessment adherence [23-25] but have limited impact on increasing smoking cessation, medication adherence, and cancer screening adherence [26-29].

Another behavioral economic strategy, information provision (IP), may improve study engagement by targeting descriptive and injunctive norms about research participation [30-32]. Descriptive norms are an individual’s perception of one’s behavior, while injunctive norms are an individual’s perception of how one approves of a behavior [30,33,34]. These norms have been shown to be predictive of risk behaviors. For instance, African American students who endorsed more permissive perceived parental injunctive norms to cannabis use were at greater risk for cannabis-related problems [35]. IP can target these norms and promote participation through a “safety in numbers” approach [30]. Specifically, this strategy can provide information that may guide an individual to feel confident in taking an action, such as telling an individual about the benefits of participating in research for themselves and others in efforts that they will then want to participate and feel secure in this decision. However, IP has demonstrated mixed results regarding its effects on patients’ knowledge of health diagnoses [36,37]. Although IP had limited effects on improving HIV knowledge [36], it did improve disease-related knowledge in patients with multiple sclerosis [37]. While IP may reform participants’ treatment preferences and lead to a greater understanding of consent and higher recruitment rates, little is known about its role in improving recruitment and retention [38,39].

Although IP and incentives are effective strategies for behavior change, they may target different aspects of motivation: IP may target intrinsic motivation (ie, the behavior itself is purposive) whereas incentives may target extrinsic motivation (ie, the prospect of financial gains motivates the behavior). Although numerous studies comparing intrinsic versus extrinsic strategies to change behavior have yielded inconsistent results, a recent meta-analysis suggested that intrinsic and extrinsic factors may act synergistically [40]. However, few studies have explicitly tested the effects of IP delivered through SMS text messaging interventions, combined with CM, to increase enrollment rates and study retention. In this randomized controlled SWAT, we evaluated the effects of IP and lotteries, alone and in combination, on study recruitment rates (ie, attending final eligibility visit) across 4 clinical trials. Secondarily, we tested effects on enrollment (eg, reaching intent-to-treat [ITT] status) and retention rates (ie, completing the study). We hypothesized that behavioral economic interventions would produce higher enrollment and retention rates than standard recruitment (SR) strategies.

## Methods

### Study Design

This pilot study is a SWAT, designed to be embedded within 4 different clinical research studies (ie, host studies) identified as having challenges with recruitment and retention rates (ClinicalTrials.gov NCT03438188, NCT02837510, NCT03169101, and NCT03384784). Although all 4 host studies recruited tobacco smokers, differences across studies are shown in Table 1.

**Table 1:** Characteristics of host trials.

ClinicalTrials.gov ID	Recruiting tobacco smokers	Recruiting people with HIV	Incorporated neuroimaging	Outcome was smoking cessation	Study duration (months)	Maximum monetary compensation^a^ (US $)
NCT03438188	✓		✓		2	514
NCT02837510	✓		✓	✓	4	810
NCT03169101	✓	✓		✓	4	440
NCT03384784	✓	✓			6	590

^a^Participants randomized to the contingency management or information provision+contingency management conditions had the opportunity to “win” an additional US $120 at each visit. However, given the very low probability of this occurring (2.15×10^–8^), the maximum compensation for each host study is listed.

### Ethical Considerations

All 4 host studies and the SWAT were approved by the institutional review board at the University of Pennsylvania (approval numbers 828958, 824061, 824860, and 828125). At the initial eligibility phone screen, participants who provided verbal consent to receive text messages were randomized to 1 of the 4 conditions for the SWAT. Written informed consent was obtained for all 4 host studies. Participants were informed that by consenting to the study, they were also consenting to receiving study communication through SMS text message. To maintain the SWAT condition blind, there were 2 versions of the consent form for each of the host studies. Participants randomized to SR, IP, or no SWAT condition received the same consent form. Participants randomized to either the CM or IP+CM condition were given a consent form that included language about participating in a lottery drawing at each study visit. All data were deidentified before analysis. All participants were provided monetary compensation for both participating in the host study and the SWAT (if randomized to the CM or IP+CM). The amounts for each study are shown in Table 1.

### Participants

Participants were recruited across various sites for each of the host trials, including infectious disease practices at the University of Pennsylvania, community-based HIV clinics, newspaper, television, and internet-based advertisements (including Craigslist, Facebook, and Twitter). To be eligible for the SWAT, all participants had to meet initial eligibility criteria for the host trial at phone screen and have SMS text messaging capabilities on their mobile device.

### Procedures

Participants completed an initial phone screen for eligibility in 1 of the 4 host studies. Eligible participants were then enrolled in SWAT using the Way to Health (W2H) platform. W2H was used for the randomization of participants into 1 of the 4 SWAT conditions and for the delivery of all SMS text messages. After enrollment into W2H, participants were randomized into 1 of 4 conditions (see below for description) and received a welcome message and baseline survey through the W2H platform. Each participant received standard text messages 1-2 days before study visits, and if randomized to the IP or information plus CM groups, participants also received targeted messages 6 days before and again 3 days before study visits. Participants were sent follow-up surveys midway through each host study and again upon completion of the study. Participants who withdrew or were lost to follow-up were sent a withdrawal survey. Survey data are not presented here.

### Measures

Demographic, smoking-, and HIV-related measures were obtained. Demographic information was ascertained. Smoking-related data (eg, smoking status, current smoking rate, and number of previous quit attempts) and the Fagerström Test for Cigarette Dependence [41] were collected at baseline. For the 2 host studies that included people with HIV, additional data included HIV viral load, CD4 lymphocyte count, and antiretroviral treatment regimen.

### Conditions

Participants were randomized to 1 of 4 conditions: (1) SR, (2) SR+IP, (3) SR+CM, and (4) SR+IP+CM (IP+CM). In the SR condition, participants received text messages 1-2 days before all study visits with relevant information about the time, date, and location of the visit, as well as contact information for study staff (“You have a study visit on [Date] at [Time]. Visit comp is US $10. Reply Y to confirm. See http://j.mp/2222222 for reminders. Reply or appt may be canceled.”).

In the IP condition, participants received personalized messages twice before each study visit (3 and 6 days before the visit). The messages were designed to target descriptive and injunctive norms regarding participating in research (eg, “[Name], wondering why you should volunteer for research? Many find it a rewarding way to advance science and be a part of a community http://j.mp/2222222.”). The messages were developed through an initial pilot study in which participants were presented with a series of messages and asked to report on the argument strength using previously validated methods [42]. Questions that were highly ranked among participants in the pilot were used as targeted messages in this study. For the list of messages used, see Multimedia Appendix 1.

In the CM condition, participants received messages about the opportunity to participate in a lottery drawing at their study visit. CM was provided in the form of a randomization table with a high proportion of numbers that correspond to “chips” with little (US $1) monetary value. Participants drew by choosing numbers between 1 and 500 upon completion of the target behavior (eg, attending an Intake visit). Upon completion of all requirements for a given visit, participants received 5 lottery “draws.” The lottery contained 500 “chips” (numbers that correspond to monetary values within a table): 250 chips had a value of US $0, 219 chips had a value of US $1, 30 chips had a value of US $5, and 1 had a value of US $100. The maximum possible earnings for 5 draws were US $120. For those who completed the study, a completion bonus was provided (ie, 5 extra draws).

In the IP+CM condition, participants received targeted messages and had the opportunity to participate in the lottery. All groups received SR and all messages were delivered through the W2H software platform. For content of all text messages and reminders, see Multimedia Appendix 1.

### Outcomes

The primary outcome was the proportion of participants who attended an intake visit to determine final eligibility. Secondary outcomes included the proportion of participants who reached ITT (as defined by each host study) and those who completed each host study. Although ITT status was defined differently across host studies, these were selected as outcomes because these are important metrics in determining recruitment success (attending eligibility visit) and the sample that will ultimately be included for analysis (ITT). For the study completers analysis, only participants who were eligible to enroll in the study were included since participants who did not meet final eligibility would not be expected to complete the study. Attending the eligibility visit was used to evaluate recruitment, ITT status was used to evaluate enrollment, and completer analysis was used to evaluate retention.

### Statistical Analysis

Baseline participant characteristics were described using chi-square, means, and SD values where appropriate. Multinomial logistic regressions were performed with condition as a between-subject effect comparing IP, CM, and IP+CM to the reference group (SR). Follow-up analyses were conducted to examine the overall effect of IP (comparing IP and IP+CM to SR) and CM (comparing CM and IP+CM to SR). For the study completers analysis, given the small sample size, the IP, CM, and IP+CM conditions were combined to compare any intervention condition to SR. Sex and host study were associated with the primary outcomes and were controlled for in subsequent analyses. Odds ratios (ORs) and 95% CIs for each pairwise comparison were obtained. To evaluate the potential cost of these recruitment strategies, we examined the average amount of money won by participants assigned to either the CM or IP+CM conditions. All analyses were performed using STATA (version 17; StataCorp) with a 5% significance level.

## Results

### Participant Characteristics

**Figure 1:**
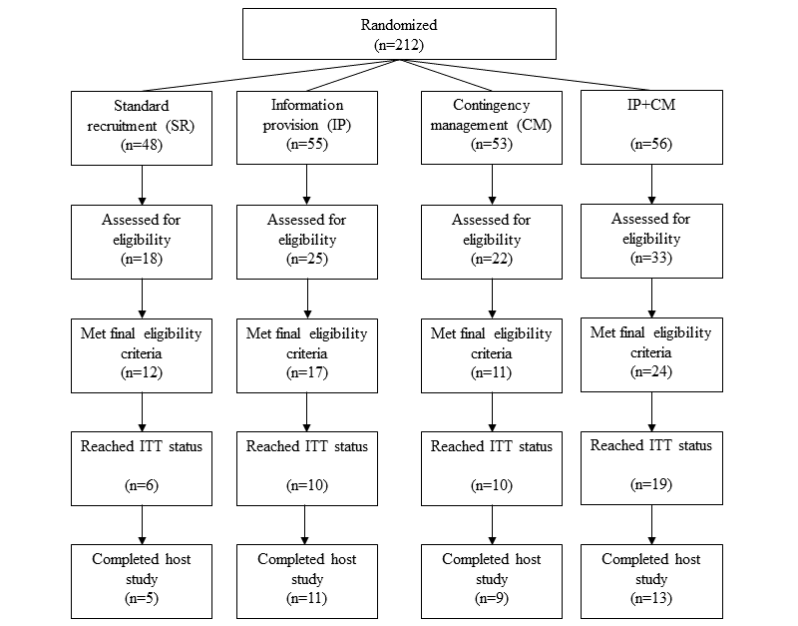
Flow of participants embedded in the study within a trial. CM: contingency management; IP: information provision; ITT; intent-to-treat; SR: standard recruitment.

A total of 212 participants were randomized and included in the analyses, as indicated in Figure 1. Most of the participants identified as Black or African American (151/212, 71.2%) and non-Hispanic (197/212, 92.9%). There was a higher proportion of participants who self-reported Hispanic ethnicity in the SR condition relative to other conditions. No other differences between conditions were found for participant characteristics, including age, race, smoking, and HIV status, as shown in Table 2. Host study characteristics are indicated in Table 1. For those randomized to either the CM or IP+CM conditions, the median amount of money earned during study participation was US $0, with a range from US $0 to US $139. The total amount of money paid to participants was US $1,556 (US $14 on average per participant).

**Table 2:** Participant characteristics (N=212).

Characteristics		Study conditions										
		IP^a^+CM^b^ (n= 56)		IP (n=55)		CM (n=53)		SR^c^ (n=48)		Total	*P* value	
**Host study ClinicalTrials.gov ID, n (%)**												.88
	NCT03169101	25 (44.6)	23 (41.8)		23 (43.4)		19 (39.6)		90 (42.5)			
	NCT03384784	13 (23.2)	14 (25.5)		12 (22.6)		15 (31.3)		54 (25.5)			
	NCT03438188	12 (21.4)	15 (27.3)		14 (26.4)		8 (16.7)		49 (23.1)			
	NCT02837510	6 (10.7)	3 (5.5)		4 (7.6)		6 (12.5)		19 (9)			
Sex (male), n (%)		35 (62.5)		34 (61.8)		39 (73.6)		33 (68.8)		141 (66.5)	.52	
Age (years), mean (SD)		48 (11.9)		52.3 (9.4)		52.1 (9.2)		48.3 (12)		50.2 (10.8)	.06	
**Race, n (%)**												.14
	White	15 (26.8)	8 (14.6)		12 (22.6)		11 (22.9)		46 (21.7)			
	African American or Black	37 (66.1)	46 (83.6)		38 (71.7)		30 (62.5)		151 (71.2)			
	Asian	0 (0)	0 (0)		1 (1.9)		1 (2.1)		2 (0.9)			
	More than 1 race	3 (5.4)	1 (1.8)		2 (3.8)		1 (2.1)		7 (3.3)			
	Unknown	1 (1.8)	0 (0)		0 (0)		4 (8.3)		5 (2.4)			
	Refused	0 (0)	0 (0)		0 (0)		1 (2.1)		1 (0.5)			
**Ethnicity, n (%)**												.03^d^
	Hispanic	2 (3.6)	3 (5.5)		1 (1.9)		7 (14.6)		13 (6.13)			
	Non-Hispanic	54 (96.4)	52 (94.6)		50 (94.3)		41 (86.4)		197 (92.9)			
	Unknown	0 (0)	0 (0)		2 (3.8)		0 (0)		2 (0.9)			
Smoking status (smoker), n (%)		50 (89.3)		47 (85.5)		44 (83)		37 (77.1)		178 (84)	.39	
HIV status (HIV+), n (%)		22 (40)		21 (39.6)		25 (50)		24 (50)		92 (44.7)	.54	

^a^IP: information provision.

^b^CM: contingency management.

^c^SR: standard recruitment.

^d^*P*<.05.

### Host Study Enrollment Rates

The proportion of participants who attended the final eligibility visit and who met ITT status is shown by condition in Figure 1. The unadjusted percentage for attendance at the final eligibility visit for each group was: IP+CM 58.9% (33/56); IP 45.5% (25/55); CM 41.5% (22/53); SR 37.5% (18/48). For the regression predicting attendance at the final eligibility visit, the IP+CM group was twice as likely to attend the final eligibility visit than the SR group (OR 2.4, 95% CI 1.1-5.2; *P=*.04). Neither the IP nor the CM condition alone were significantly different from the SR condition (*P*=.41 and *P=*.74, respectively). In the follow-up model, those who received the IP condition (controlling for CM) were slightly more likely to reach and attend the final eligibility visit (OR 1.7, 95% CI 1.0-2.9; *P*=.07); CM did not have a significant effect (*P*=.20).

The unadjusted percentage for reaching ITT status for each group was: IP+CM 33.9% (19/56); IP 18.2% (10/55); CM 18.9% (10/53); SR 12.5% (6/48). Females were more likely to reach ITT status than males (OR 2.7, 95% CI 1.2-5.7; *P*=.01). The IP+CM group was significantly more likely to reach ITT status than the SR condition (OR 3.9, 95% CI 1.3-11.1; *P*=.01). Neither the IP nor the CM condition alone were significantly different from the SR condition (*P=*.48 and *P=*.28, respectively). In contrast to the model predicting final eligibility, the follow-up model suggested that, when controlling for IP, CM increased the likelihood of reaching ITT status (OR 2.3, 95% CI 1.1-4.7; *P*=.03).

### Host Study Retention Rates

The proportion of participants completing the study is shown by condition in Figure 1. Females were slightly more likely than males to complete the host study (OR 3.0, 95% CI 0.9-9.7; *P*=.07). Although the proportion of study completers who were randomized to an active intervention (33/52, 63.5%) was higher than those who received only SR (5/12, 41.7%), the effect was not significant (*P*=.26).

## Discussion

### Overview

Clinical trials are essential for creating evidence-based interventions but have continuously faced challenges from underenrollment, financial costs, and retaining participants. This randomized controlled SWAT sought to evaluate the efficacy of 2 strategies grounded in behavioral economics to promote enrollment and engagement in clinical trials. We found that our behavioral economic strategies, IP and CM combined, increased study enrollment and retention. Furthermore, we found that participant characteristics (eg, gender) may also be related to study participation. This study provides insight into using evidence-based, cost-effective SMS text messaging interventions that incorporate behavioral economic strategies to address a common challenge when conducting clinical trials.

Previous research suggests that IP and CM are valuable strategies when used separately, in recruitment and retention efforts [20,38,39]. We extend these findings and demonstrate that the combination of IP and CM substantially improved the likelihood that a participant would attend an eligibility visit and reach ITT status. Neither strategy alone had a significant effect. The increase in enrollment rates among those receiving both strategies may be due in part to differences in the type of motivation targeted by each strategy. Preliminary studies have shown that motivation may be one feature by which these techniques can improve targeted behavior, like HIV health-related outcomes [43,44]. Indeed, a 2018 study found that incorporating financial incentives helped increase urine screening adherence when internal motivation was relatively low [43].

Evidence from this study also reveals participants who only received either IP or CM did not differ from those who only received standard messages. Although our follow-up analyses show that IP (alone or in combination with CM) may have driven the effect on final eligibility, whereas CM (alone or IP+CM) increased the likelihood of reaching ITT status, these analyses should be interpreted cautiously due to our relatively small sample size. Our targeted messages encouraging participant involvement may have promoted feelings that participation in research is socially valued [45]. Moreover, understanding the beneficial effects of research on society may facilitate research engagement, in turn, producing higher enrollment rates. As our data suggest, explicitly communicating the value of participating in research could be a core component of IP. Indeed, previous work has demonstrated IP to be an effective strategy for communicating knowledge of the study protocol and eliciting participant preferences for treatment [39,46,47].

Incorporating CM through a lottery increased enrollment rates when combined with IP. These findings provide support for our initial scientific premise: targeting both intrinsic motivation (through IP) and extrinsic motivation (through CM) would have the greatest effect on enrollment rates. Many studies cite concerns with incorporating financial incentives, such as lack of funding [48-50]. Our data suggest that financial incentives through a lottery-based system can be a cost-effective way to target extrinsic motivation. Employing these 2 behavioral economic strategies together was not more expensive than the CM-only group and cost only US $14 per participant, on average. Thus, this is a feasible and cost-effective method for improving recruitment. Indeed, a Cochrane survey found that monetary incentives increased participant response to both postal and electronic questionnaires, improving study retention [50]. Similarly, other studies have found increased retention rates using monetary incentives [51,52]. Our data suggest that implementing messages on IP and using lottery incentives as a form of CM are useful strategies for improving recruitment and retention in clinical research studies.

Despite the observed increase in enrollment rates, our findings indicate no significant differences in completion rates between the 4 conditions. However, completion rates were low overall, which may partially explain this null result. We did find that women were more likely to achieve ITT status and complete the study compared to men. Although the mechanism underlying this gender difference is unknown, this information may guide recruitment strategies for clinical research. Given the higher attrition rates among men, it may be important to overenroll men and focus on retention. Overall rates of research participation are lower among women, suggesting the need for targeted strategies to increase initial interest in participation.

### Limitations

Some limitations should be acknowledged when interpreting our findings. First, while SMS text messaging interventions have been incorporated in numerous studies to promote participant engagement, some implementation barriers to SMS text messaging interventions remain [53,54]. Although fewer than 5% (10/222) of participants were excluded from the SWAT because of phone incompatibility with SMS text messaging, changes in data plans and phone numbers were common, making continuity of communication difficult at times. Second, sample size may have limited our ability to detect differences between conditions, particularly for study completion rates. Given that this study is a pilot study, an a priori power analysis was not conducted. Larger studies are needed to evaluate individual components (eg, IP vs CM) as well as moderators of the interventions’ effects. Third, this study did not employ an attention control condition to account for the extra messages participants in the IP, CM, and IP+CM groups received. However, participants in each of the active conditions received the same number of text messages. Thus, it is unlikely that our finding that individuals in the IP+CM condition had the highest recruitment and enrollment rates was due to the higher number of messages sent. Fourth, recruitment for all 4 host studies was interrupted by the COVID-19 pandemic; this was particularly true for the 2 studies that involved functional magnetic resonance imaging. All studies pivoted to remote procedures when feasible, and it is unclear how this may have impacted study participation.

### Conclusions

By using a rigorous, randomized control design, this study advances the literature on behavioral economic strategies to promote recruitment and retention in clinical trials. Our data suggest that combining IP and CM strategies may motivate participants to participate in research. Furthermore, each behavioral economic strategy represents a cost-effective and evidence-based technique that enhances study enrollment. Future studies should evaluate the efficacy of these strategies across different samples and trial designs and further analyze how potential moderators (eg, financial stress) or core components of each strategy (eg, message content) may impact the efficacy of these strategies.

## Appendix

Multimedia Appendix 1 Text message content.

## Abbreviations

CM: contingency management
IP: information provision
ITT: intent-to-treat
OR: odds ratio
SR: standard recruitment
SWAT: study within a trial
W2H: Way to Health

## Data Availability

The deidentified data sets generated and analyzed during this study are available upon request by contacting the corresponding author.
