# Supplementary material for: Behavioral Economic Strategies to Improve Enrollment Rates in Clinical Research: Embedded Recruitment Pilot Trial
Source: JMIR Form Res. 2023 Jul 21;7:e47121. doi: 10.2196/47121 (PMC10403799; doi:10.2196/47121)
Supplement: Multimedia Appendix 1 [file formative_v7i1e47121_app1.docx]

Supplementary Information for Behavioral Economic Strategies to Improve Enrollment Rates in Clinical Research: Embedded Recruitment Pilot Trial

Control/Standard SMS messages 1-3

**C1** You have a study visit on [Date] at [Time]. Visit comp is $10. Reply Y to confirm. See [study website link] for reminders. Reply or appt may be canceled.

**C2** [Name], your appt for the research study is on [Date] at [Time]. Continue to smoke as usual. Total comp for 4-day period is up to $165. Reply Y to confirm.

**C3** As a thank you for donating your time, you will automatically be entered into a lottery for a chance to win $250 just for attending an intake visit!

Targeted SMS messages

Recruitment phase 1-7

**REC4** Research is an important part of making scientific advancements. When you donate your time you are helping to move science forward!

**REC6** Research volunteers often feel proud once they complete a study. By giving your time to research you will become part of the scientific community!

Retention phase 1-14

**RET1** [Name], you are a key part of our research team. By volunteering, you are helping us understand smoking behavior and develop quit treatments!

**RET2** NAME, thank you for enrolling in our study. We truly appreciate the time and effort you are dedicating to this research!

**RET5** You have completed X visits as a part of this research study, which is a great achievement! We appreciate your continued participation!

**RET6** You are half way through the study, only X more visits to go. Thank you for your dedication to the study!

**RET7** The data you provide as a study participant is unique and important. We couldn’t run the study without volunteers like you. Thank you for your efforts!

**RET9** [NAME], We couldn’t do this research study without participants like you! Thank you for your continued interest and dedication to helping us understand smoking behaviors and develop quit treatments!

**RET10** [NAME], You have completed the research study with us! Thank you for all of your time and effort. We are now one step closer to better understanding smoking behaviors and developing quit treatments!

**RET11** [NAME], have you told your friends and family about your contribution to our research study? It’s a great way to spread the word about participating in research!

**RET12** [NAME], thank you for your continued participation in our research study. Your effort is helping us learn the best ways to help others like you quit smoking!

**RET13** [NAME], did you know that by participating in research you are helping scientists discover better quit smoking treatments? Thank you for being part of our study!

**RET14** [NAME], we appreciate the time and effort you are putting into the research study. Your contribution to smoking research is valuable and important!
